# Presentation of an Immunodominant Immediate-Early CD8+ T Cell Epitope Resists Human Cytomegalovirus Immunoevasion

**DOI:** 10.1371/journal.ppat.1003383

**Published:** 2013-05-23

**Authors:** Stefanie Ameres, Josef Mautner, Fabian Schlott, Michael Neuenhahn, Dirk H. Busch, Bodo Plachter, Andreas Moosmann

**Affiliations:** 1 Clinical Cooperation Group Immunooncology, Department of Medicine III, Klinikum der Universität München, and Department of Gene Vectors, Helmholtz Zentrum München, Munich, Germany; 2 DZIF – German Center for Infection Research, Munich, Germany; 3 Clinical Cooperation Group Pediatric Tumor Immunology, Helmholtz Zentrum München, and Children's Hospital, Technische Universität München, Munich, Germany; 4 Institute for Medical Microbiology, Immunology and Hygiene, Technische Universität München, Munich, Germany; 5 Clinical Cooperation Group Immune Monitoring, Helmholtz Zentrum München and Technische Universität München, Munich, Germany; 6 Institute for Virology, University Medical Center, Johannes-Gutenberg-Universität Mainz, Mainz, Germany; University of Alabama at Birmingham, United States of America

## Abstract

Control of human cytomegalovirus (HCMV) depends on CD8+ T cell responses that are shaped by an individual's repertoire of MHC molecules. MHC class I presentation is modulated by a set of HCMV-encoded proteins. Here we show that HCMV immunoevasins differentially impair T cell recognition of epitopes from the same viral antigen, immediate-early 1 (IE-1), that are presented by different MHC class I allotypes. In the presence of immunoevasins, HLA-A- and HLA-B-restricted T cell clones were ineffective, but HLA-C*0702-restricted T cell clones recognized and killed infected cells. Resistance of HLA-C*0702 to viral immunoevasins US2 and US11 was mediated by the alpha3 domain and C-terminal region of the HLA heavy chain. In healthy donors, HLA-C*0702-restricted T cells dominated the T cell response to IE-1. The same HLA-C allotype specifically protected infected cells from attack by NK cells that expressed a corresponding HLA-C-specific KIR. Thus, allotype-specific viral immunoevasion allows HCMV to escape control by NK cells and HLA-A- and HLA-B-restricted T cells, while the virus becomes selectively vulnerable to an immunodominant population of HLA-C-restricted T cells. Our work identifies a T cell population that may be of particular efficiency in HCMV-specific immunotherapy.

## Author Summary

Human cytomegalovirus is very widespread. It cannot be eliminated from the body. The health of its host depends on immune responses that are maintained for a lifetime. Virus-specific CD8-positive T cells recognize and kill infected cells before they can produce more virus. Infected cells are recognized because they display MHC class I molecules that have bound a peptide derived from a viral protein. The three most relevant human MHC I genes (HLA-A, -B, -C) occur in many allelic variants that present different viral peptides to different T cells. HCMV encodes several molecules that interfere with stability or localization of HLA class I, but it is unknown how this affects recognition by T cells that recognize infection through different HLA molecules. We show here for one viral antigen, IE-1, that viral interference with T cell recognition strongly depends on the identity of the HLA molecule: HLA-A- and HLA-B-restricted T cells are fully inhibited, but HLA-C-restricted T cells that target an epitope from the same antigen recognize and kill infected cells with high efficiency. Because this population of HLA-C-restricted T cells has superior antiviral function and elevated frequency in the blood, it may be particularly suitable for immunotherapy.

## Introduction

Human cytomegalovirus (HCMV) latently infects a majority of humans for their lifetime. Infection is usually asymptomatic, but can cause severe morbidity and mortality in immunocompromised patients and after congenital or neonatal infection [1]. Cellular immunity, and in particular the virus-specific CD8+ T cell response, is of vital importance for controlling the virus [2]. For example, after allogeneic stem cell transplantation, virus-specific CD8+ T cells are associated with protection from HCMV disease [3], and specific immunity in patients can be reconstituted by adoptive transfer of virus-specific CD8+ T cells [4]–[6]. Congenital HCMV infection has a higher frequency of causing harm when a non-immune mother acquires the virus for the first time during pregnancy [7], suggesting that pre-established maternal immunity is partially protective. However, neither HCMV-specific adoptive T cell therapy nor HCMV-specific vaccines [8], [9] have moved beyond the stage of clinical testing.

Various HCMV antigens are targeted by CD8+ T cells [10], [11], but only a small number of antigens elicit immunodominant responses in a majority of healthy carriers [11], [12]. Among these, the 72-kDa immediate-early 1 protein (IE-1; UL123) deserves attention. IE proteins are the first to be expressed in viral replication [13] and initiate viral gene expression leading to virus production. Therefore, IE-1-specific CD8+ T cells could potentially control viral reactivation from latency before virus is produced [14], [15]. In murine CMV infection, IE-1-specific CD8+ T cells are protective [16], [17]. Although clinical responses were also observed after transfer of T cells specific for other HCMV antigens, in particular pp65 [4], [6], CD8+ T cells specific for IE-1 are associated with protection from viral disease or reactivation in patients after different types of transplantation [18], [19]. A variety of IE-1 epitopes restricted through different MHC class I (HLA-A and B) allotypes are recognized by CD8+ T cells [10], [20], [21]. Therefore, IE-1 seems an attractive target of HCMV-specific immunotherapy.

In spite of these arguments, the role of IE-1-specific CD8+ T cells in control of HCMV has remained controversial, because it was observed that their recognition of infected cells is strongly inhibited [22] by a set of HCMV-encoded proteins that prevent the presentation of viral peptides by MHC class I on the cell surface [12], [23]. In contrast, others have reported that IE-1-specific T cells efficiently recognize infected cells [24], although it is not known which epitopes were responsible for this recognition. Several immunoevasins encoded in the US2-11 region of the HCMV genome interfere with antigen presentation on MHC class I by preventing peptide transport (US6), retaining MHC molecules in the endoplasmic reticulum (US3), or targeting them for cytoplasmic degradation (US2, US11) [25]–[27]. Biochemical studies have indicated that viral immunoevasins downregulate the expression of different MHC class I allotypes to different degrees [28], [29]. However, it is not known whether such allotype-specific variations in viral immunoevasion affect recognition of infected cells by specific CD8+ T cells. For developing T cell therapies and vaccines, the identification of HCMV peptide/MHC class I complexes that are functionally recognized in spite of viral immunoevasion would be important. To address these issues, we analyze here the effect of HCMV immunoevasins on the recognition of infected cells by IE-1-specific CD8+ T cell clones of different HLA restrictions. Our results show that antigen presentation to T cell clones representing a newly identified, immunodominant HLA-C-restricted CD8+ T cell population, but not to a panel of HLA-A- and HLA-B-restricted T cell clones, is selectively preserved in infected cells. Conversely, killing of infected cells by NK cells is inhibited by the same HLA-C allotype. Thus, HCMV immunoevasion appears to exempt this HLA-C allotype in order to avoid control by NK cells. As a consequence, certain HLA-C-restricted CD8+ T cells efficiently recognize IE-1 presented by infected cells. Such effectors may be preferable targets of HCMV vaccine and immunotherapy.

## Results

### Identification of HLA-C-restricted IE-1-specific CD8+ T cells

**Figure 1:**
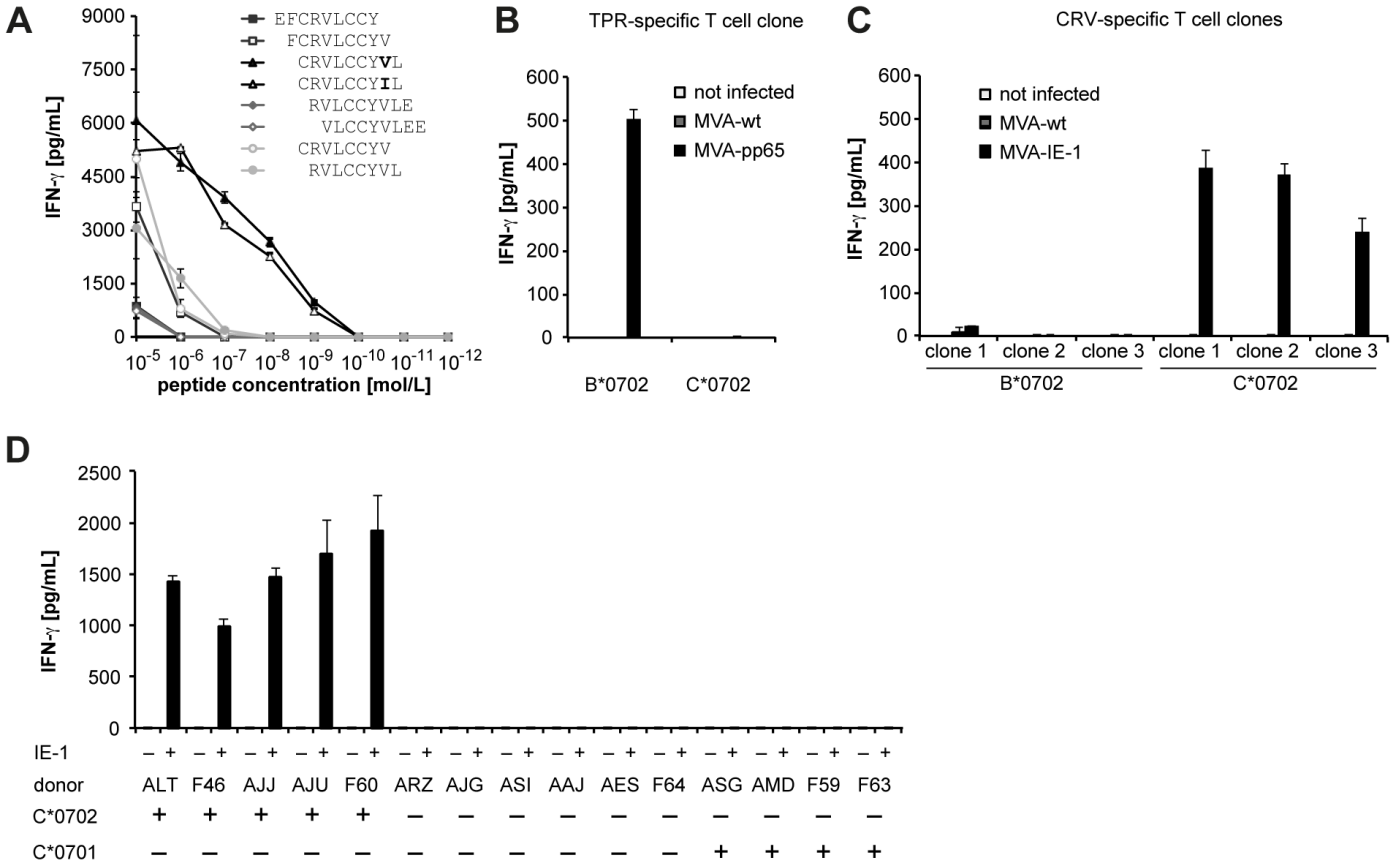
Identification of HLA-C*0702-restricted IE-1-specific CD8+ T cells. (A) Reactivity of T cell clone ALT#127 to titrated IE-1 peptides in IFN-γ ELISA (20 000 clonal T cells and 50 000 autologous peptide-loaded CD40-stimulated B cells per well in duplicates). (B, C) HLA restriction of the IE-1 CRV epitope. WI-38 fibroblasts were transfected with HLA-B*0702 or C*0702 and infected with MVAs encoding pp65 (B) or IE-1 (C), alongside with negative controls (no infection and wild-type (wt) MVA infection). Cells were then coincubated overnight with T cell clone F37#5 specific for pp65/TPR/B*0702 (B) or IE-1/CRV-specific T cell clones (C) from 3 different donors (AJJ, AJU and ALT). For each condition, 10 000 fibroblasts and 10 000 T cells were coincubated in 3 replicates. Supernatants were analyzed in an IFN-γ ELISA. (D) The reactivity of T cell clone AJU#90 to IE-1-expressing or control mini-LCLs from donors with various HLA types was tested in IFN-γ ELISA (10 000 T cells and 20 000 mLCLs in two replicates). For full HLA class I types, see Table S1.

To obtain IE-1-specific CD8+ T cell lines containing various epitope specificities and HLA restrictions *in vitro*, we restimulated PBMCs from randomly chosen HCMV-positive donors with IE-1-expressing mini-lymphoblastoid cell lines (mLCLs), as previously reported for pp65 [30], [31]. The resulting polyclonal T cell lines were subjected to limiting dilution to obtain CD8+ T cell clones. The IE-1 peptides recognized by these T cell clones were identified using an overlapping 15-mer peptide library covering the IE-1 sequence [20]. Many CD8+ T cell clones from HLA-B*0702/C*0702-positive carriers were specific for a nonameric IE-1-derived peptide CRVLCCYVL (abbreviated CRV, amino acids 309–317) (Figure 1A). A variant peptide with the sequence CRVLCCYIL, which is present in some HCMV strains including Toledo and TB40E, was recognized with similar affinity (Figure 1A). The CRV peptide was described earlier to be restricted by HLA-B*07 [20]. Because this peptide lacks a proline in position two, which is required for B*07-restricted peptides [32], we tested the possibility that the peptide was instead restricted through HLA-C*0702, an allele generally associated with HLA-B*0702 in Europeans [33]. WI-38 fibroblasts were transfected with HLA-B*0702 or -C*0702 and infected with pp65- or IE-1-encoding modified vaccinia virus Ankara (MVA). The HLA-B*0702-restricted pp65-derived epitope TPR [34] was recognized by a specific T cell clone in HLA-B*0702-transfected but not in HLA-C*0702-transfected targets (Figure 1B). Conversely, CRV-specific T cell clones from different donors recognized IE-1 in the context of HLA-C*0702, but not HLA-B*0702 (Figure 1C). CRV-specific T cells recognized IE-1-expressing cells from various C*0702-positive donors, but not cells from C*0701-positive or other donors (Figure 1D). Thus, CRV is restricted through HLA-C*0702, but not HLA-C*0701. This explains earlier observations that responses to the CRV peptide were consistently found in B*07/C*07-positive but not in B*08/C*07-positive donors [20], because the former generally carry HLA-C*0702, the latter HLA-C*0701 [33] (compare Table S1). These data show that HLA-C-restricted IE-1-specific T cells are part of the T cell repertoire of HCMV carriers.

### Immunodominance of HLA-C*0702-restricted HCMV-specific T cells

**Figure 2:**
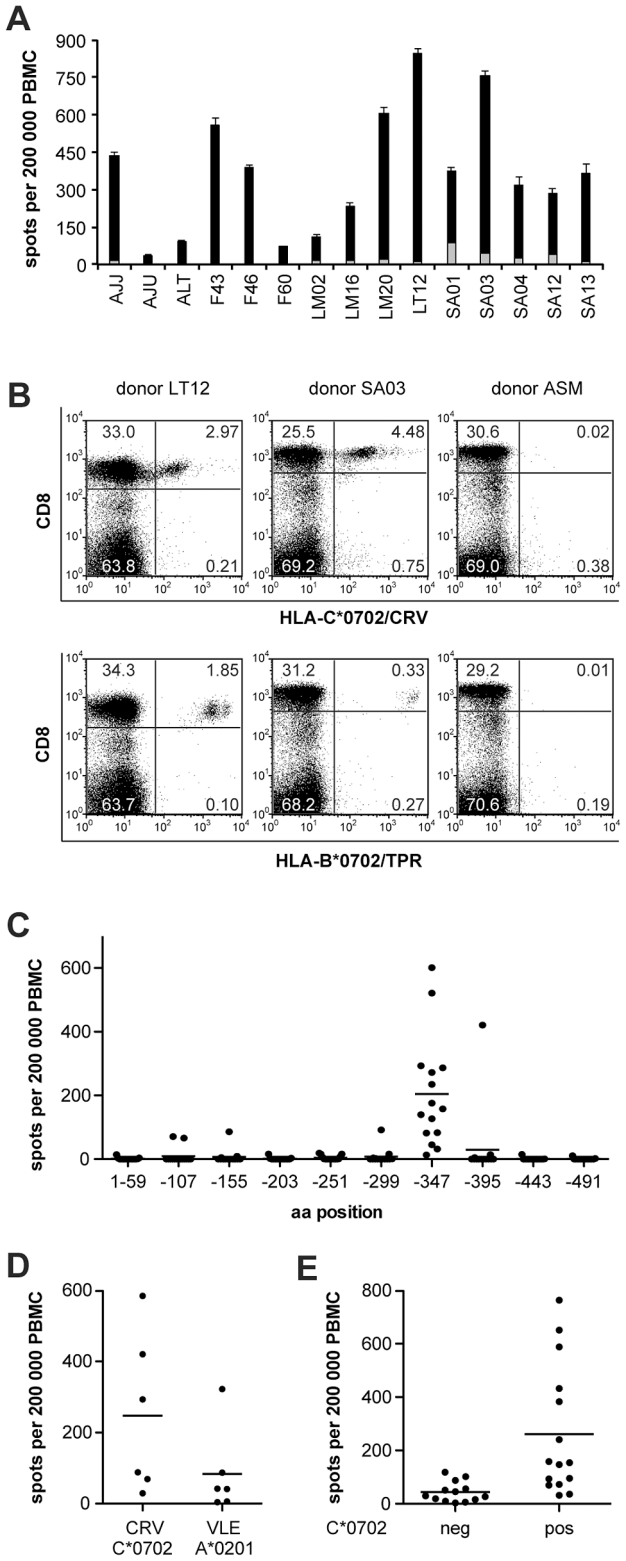
Immunodominance of CRV/C*0702-specific T cells. (A) Frequencies of CRV-specific T cells in 15 HLA-C*0702-positive blood donors, tested with the CRV nonameric peptide in ELISPOT assays. Black parts of bars indicate CRV-specific signal, grey parts indicate background (no peptide). (B) Specific T cells in PBMCs of HLA-C*0702 carriers were quantified by fluorescent staining with HLA-C*0702/CRV streptamer or HLA-B*0702/TPR pentamer and anti-CD8 antibody. Donors LT12 and SA03 are HCMV-seropositive, donor ASM is HCMV-seronegative. (C) Distribution of T-cell targets within the IE-1 sequence for 15 HLA-C*0702-positive donors, tested with overlapping peptides covering the entire IE-1 sequence of strain AD169. The 120 peptides were divided into 10 subpools, each comprising 12 successive 15-mer peptides with an overlap of 11 amino acids. The C-terminal amino acid position of each subpool is indicated. (D) Frequencies of CRV/C*0702-specific and VLE/A*0201-specific T cells in HLA-C*0702/A*0201-positive donors (n = 6). (E) Comparison of IE-1-specific T cell frequencies in C*0702-negative (n = 13) vs. C*0702-positive (n = 15) donors. (A, C–E) IFN-γ ELISPOT assays were performed with 200 000 peptide-loaded PBMCs in each well and with 2–4 replicates per condition.

We analyzed the repertoire of IE-1-specific CD8+ T cells in healthy HCMV carriers. T cells specific for the HLA-C*0702-restricted peptide CRV could be directly detected in PBMCs of 15/15 HLA-C*0702-carrying donors by ELISPOT (Figure 2A), with a mean frequency of 345 specific spots per 200 000 PBMCs (range 29–837). Multimer staining (Figure 2B) showed that CRV/C*0702-specific T cells were more frequent than TPR/B*0702-specific T cells, a known immunodominant pp65 specificity [35]. Within IE-1, the region containing CRV was dominantly recognized (Figure 2C), and CRV was the dominant IE-1 epitope in 11 out of 15 donors (data not shown). In donors who were HLA-A*0201 and HLA-C*0702-positive, CRV-specific T cells were on average 3-fold more frequent than T cells specific for the dominant A*0201-restricted epitope VLEETSVML (VLE) [21] (Figure 2D). The total number of IE-1-reactive T cells was higher in HLA-C*0702-positive donors than in HLA-C*0702-negative donors (Figure 2E), mainly as a result of the high frequencies of CRV-specific T cells.

### CRV/C*0702 is recognized on infected cells by specific T cells

**Figure 3:**
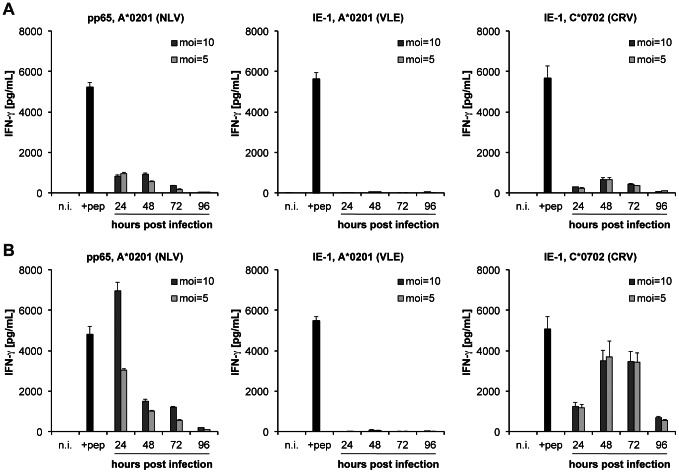
Time course of pp65- and IE-1-specific CD8+ T cell recognition of HCMV-infected fibroblasts. pp65 and IE-1 T cell epitopes were analyzed for their HLA-A*0201 or -C*0702-restricted presentation to T cell clones at different time points post infection. MRC-5 fibroblasts (HLA-A*0201, C*0702) were not treated (A) or were treated (B) with IFN-γ for 72 hours prior to infection with HCMV AD169 at an moi of 5 or 10. At the indicated time points post infection, 10 000 fibroblasts were incubated with 10 000 T cells for 16–18 hours before measuring antigen-specific IFN-γ secretion by ELISA assay. Cells that were not infected (n.i.) or were peptide-loaded 48 hours post infection (+pep) served as controls. Data are shown as mean+SD of triplicate samples. One of three independent experiments with clones ALT#21 NLV, F61#38 VLE and AJJ#7 CRV is shown, representing experiments with a total of 3 NLV-, 6 VLE- and 12 CRV-specific T cell clones, from 3 different donors for each specificity.

The recognition of previously studied IE-1 epitopes by specific T cells in HCMV-infected fibroblasts is severely suppressed [22] due to the action of viral immunoevasive proteins that interfere with MHC class I presentation [23], but there is evidence suggesting that this may not uniformly be true for all IE-1 epitopes [21], [24]. We investigated whether recognition of infected fibroblasts by CRV/C*0702-specific T cells was similarly impaired. T cell clones specific for A*0201-restricted epitopes NLV from pp65 and VLE from IE-1 were tested in parallel. Before infection with HCMV strain AD169 at a multiplicity of infection (moi) of 5 or 10, fibroblasts were cultivated in standard culture medium (Figure 3A) or IFN-γ-supplemented medium (Figure 3B). Recognition of pp65/NLV/A*0201 was strong in the early stages of infection and decreased over time, which is typical for abundant structural antigens such as pp65 [36] that reach the infected cell as a part of the virion. Recognition of the A*0201-restricted IE-1 epitope VLE was abolished at all times in both conditions. In contrast, the HLA-C*0702-restricted IE-1 epitope CRV was recognized on infected fibroblasts at all times, with a maximum on days 2 and 3. The kinetics of CRV presentation closely followed, with a 1-day delay, the expected kinetics of IE-1 expression in infected fibroblasts [37]. Upon pretreatment with IFN-γ, recognition of the pp65 epitope NLV and IE-1 epitope CRV in infected cells was further increased without qualitatively altering the observed recognition kinetics, while recognition of the IE-1 VLE epitope was not rescued by IFN-γ pretreatment. Thus, the CRV/C*0702 epitope escaped the immunoevasive mechanisms that prevented the presentation of another IE-1 epitope in infected cells.

### HCMV immunoevasins interfere with T cell recognition in an allotype-specific manner

**Figure 4:**
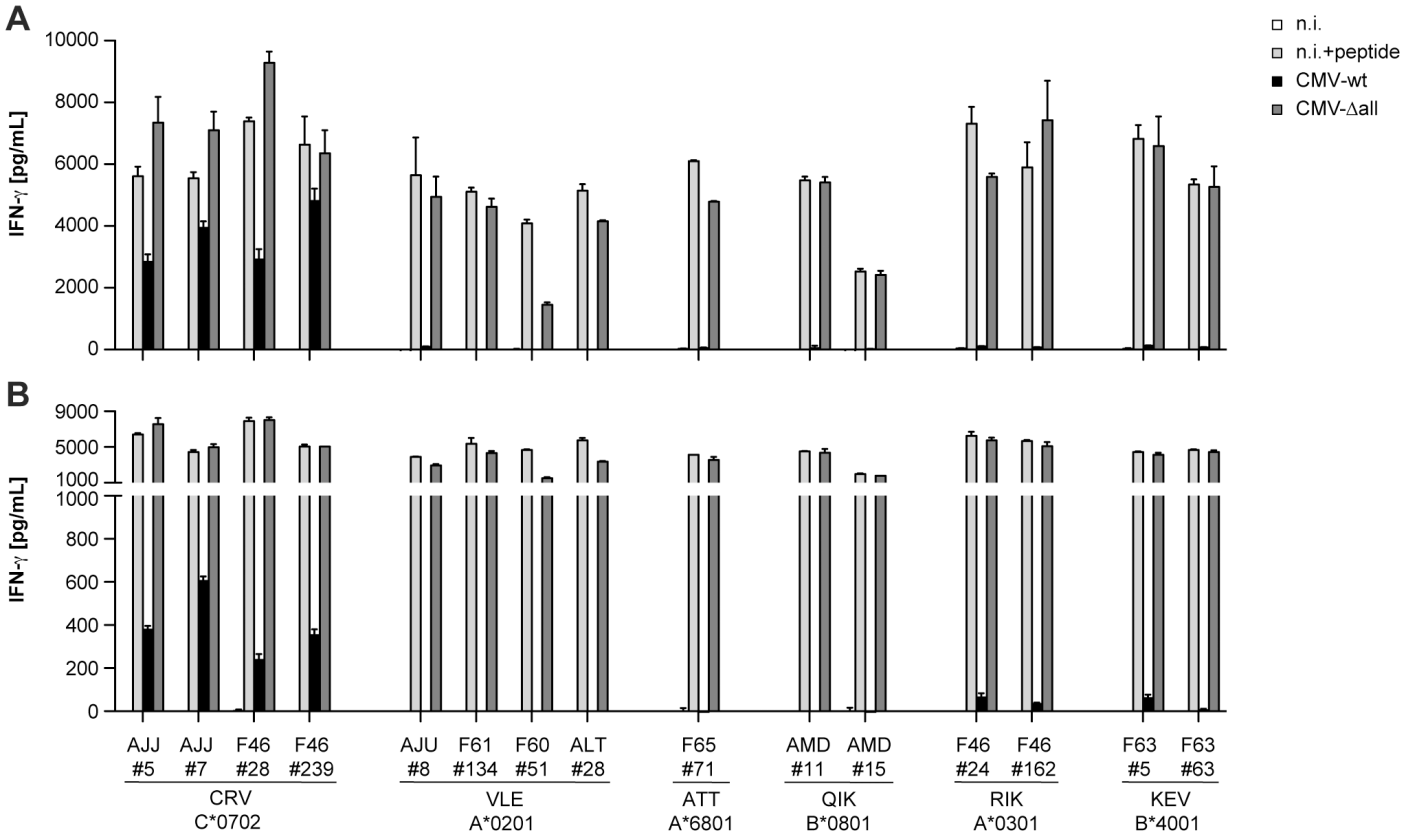
Collective impact of HCMV immunoevasins on the recognition of IE-1 T cell epitopes with different HLA restrictions. Fibroblasts were infected with HCMV AD169 (CMV-wt) or an AD169 variant lacking the four immunoevasins US2, 3, 6, and 11 (CMV-Δall). Recognition by various IE-1 epitope-specific T cell clones with the indicated specificities was analyzed. To cover every required HLA allotype, three primary fibroblast cell lines were used: MRC-5 (C*0702, A*0201), WI-38 (A*6801, B*0801), BFF2 (A*0301, B*4001). Fibroblasts were precultured in the presence (A) or absence (B) of IFN-γ for 72 hours, then infected at moi = 5 and cocultivated with T cells at 48 hours post infection (10 000 fibroblasts and 10 000 T cells per well). IFN-γ secretion was measured by ELISA. Targets included cells that were not infected (n.i.) or peptide-loaded (n.i. +peptide). One to four representative T cell clones of each specificity were analyzed. Data are shown as mean+SD of triplicate samples. One representative of three independent experiments is shown.

We studied the allotype-specific impact of HCMV immunoevasins, employing a larger panel of IE-1-specific CD8+ T cell clones with various HLA restrictions. Target cells were infected with wild-type HCMV or an HCMV derivative deleted for the four immunoevasins US2, US3, US6, and US11 (CMV-Δall). Our analyses included IE-1-specific CD8+ T cell clones restricted through HLA-A*0201, A*0301, A*6801, B*0801, B*4001, and C*0702. Among 15 T cell clones with these 6 different HLA restrictions, only the four C*0702-restricted clones showed a distinct reactivity to HLA-matched fibroblasts infected with wild-type HCMV (Figure 4). Although recognition of HLA-C*0702-restricted antigen was weaker when the fibroblasts were not pretreated with IFN-γ (Figure 4B) than after such a pretreatment (Figure 4A), preferential recognition of the HLA-C*0702-restricted epitope as compared to all HLA-A- and B-restricted epitopes was seen for both conditions. In contrast, infection with the CMV-Δall virus was recognized by most T cell clones with similar intensity irrespective of HLA restriction, and T cell recognition after CMV-Δall infection was similar to recognition after exogenous loading with peptide in each case. The observation that all peptides were maximally presented after CMV-Δall infection suggested that escape of HLA-C-restricted presentation was not caused by a selective overabundance of specific peptide that led to a saturation of immunoevasins, as proposed for some epitopes in the mouse model [38], but was due to the allotype-specific action of HCMV immunoevasins. Thus, viral immunoevasins US2, 3, 6, and 11 completely blocked antigen presentation by each of the HLA-A and HLA-B alleles tested, but not by HLA-C*0702.

**Figure 5:**
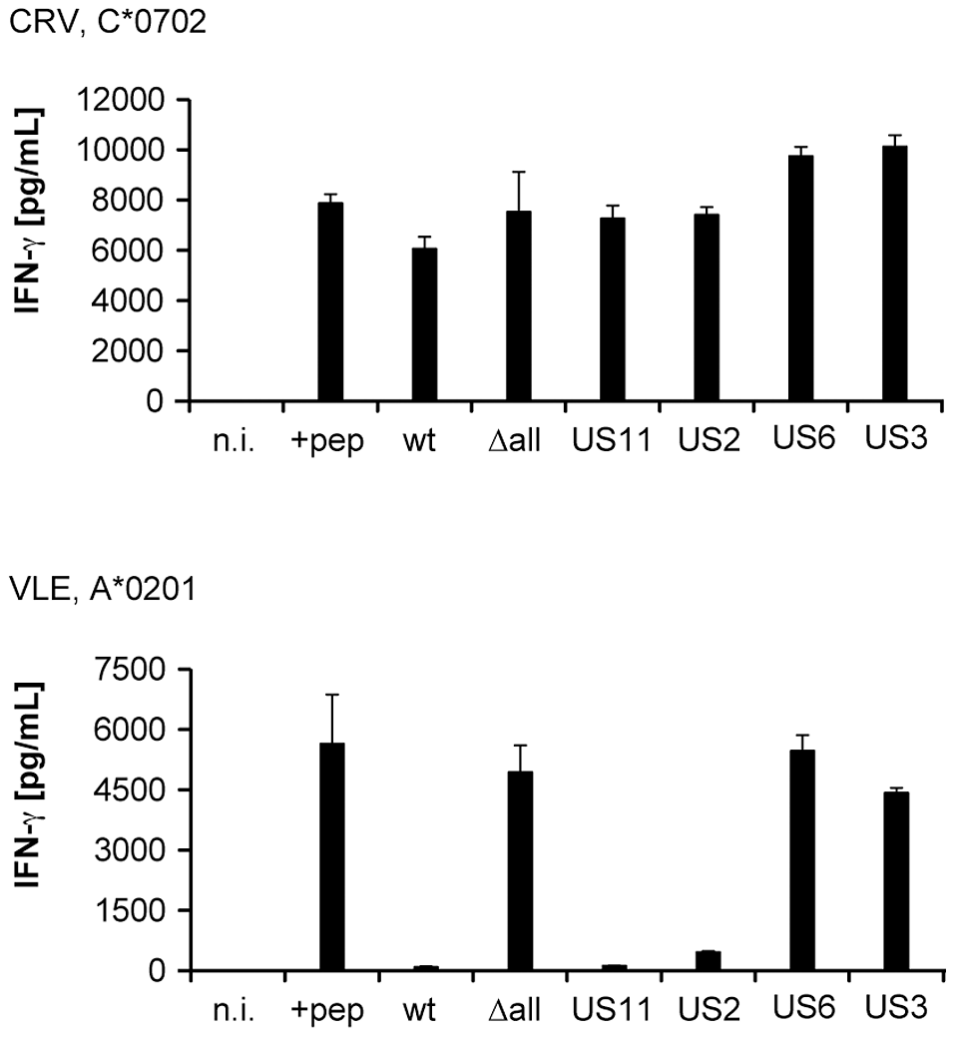
Impact of individual HCMV immunoevasins on the recognition of IE-1 T cell epitopes. MRC-5 fibroblasts were infected with HCMV strain AD169 (wt), with AD169 derivative viruses that expressed only one of the four immunoevasins US2, US3, US6, or US11 as indicated, not infected (n.i.) or peptide-loaded (+pep), and their recognition by T cell clones specific for the IE-1 epitopes CRV/C*0702 and VLE/A*0201 was analyzed. Before infection, fibroblasts were precultured with IFN-γ for 72 hours, then infected at moi = 5 and cocultivated with T cells at 48 hours post infection (10 000 fibroblasts and 10 000 T cells per well). IFN-γ secretion was measured by ELISA. Data are shown as mean+SD of triplicate samples. Representative data are shown for one of 10 CRV-specific clones and one of 4 VLE-specific clones, assayed in two independent experiments.

Next, we analyzed the role of individual HCMV immunoevasins in modulating the presentation of different IE-1 epitopes by infected cells. We infected fibroblasts with a set of HCMV mutant viruses that expressed only one of the four immunoevasins US2, 3, 6, and 11, but were deleted for the other three [37], [39], [40], and measured antigen presentation to CD8+ T cell clones specific for the IE-1 epitopes CRV/C*0702 and VLE/A*0201 (Figure 5). In this experiment, fibroblasts were pretreated with IFN-γ before infection. Under these conditions, each of the immunoevasins US2 and US11 was capable of downregulating the presentation of the VLE/A*0201 epitope to near completion, whereas US3 and US6 did not have a major impact on the presentation of this epitope. In contrast, none of the individual immunoevasins had a strong inhibitory effect on the presentation of CRV/C*0702; a slight effect of US2 and US11 seemed to be present, and the action of these two immunoevasins may be mainly responsible for the observed small reduction of CRV presentation after CMV wild-type infection. This experiment showed that impairment of antigen presentation by each of US2 and US11 is strongly allotype-dependent: HLA-A*0201 is very efficiently targeted by these immunoevasins, HLA-C*0702 is targeted to a very limited extent.

**Figure 6:**
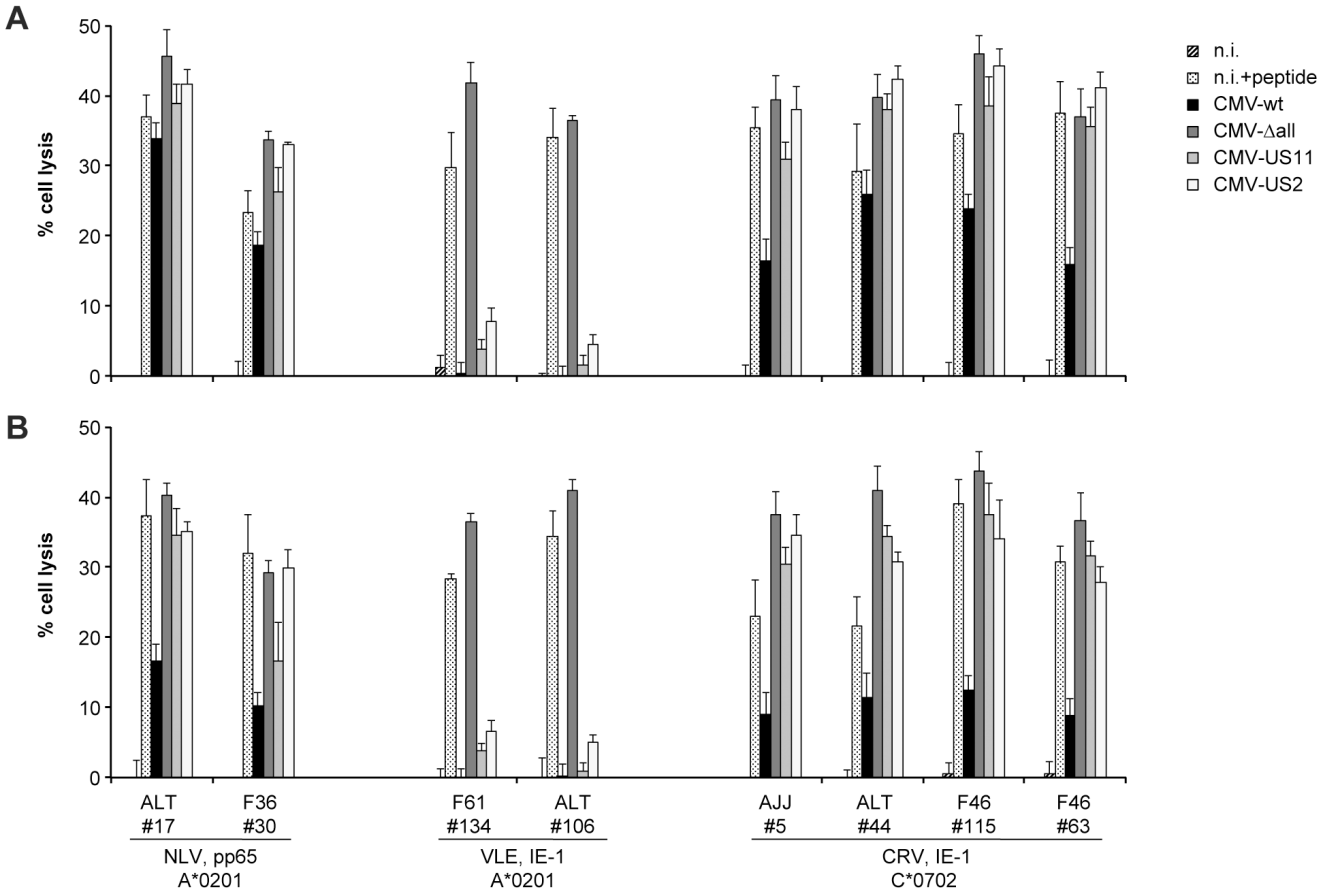
Effect of HCMV immunoevasins on epitope-specific T cell cytotoxicity. MRC-5 fibroblasts (HLA-A*0201, C*0702) were preincubated in medium with (A) or without (B) IFN-γ for 72 hours before infection at moi = 5 with AD169 (CMV-wt) or its derivatives CMV-Δall (ΔUS2/3/6/11), CMV-US11 (ΔUS2/3/6) or CMV-US2 (ΔUS3/6/11). Cytotoxicity was determined at 48 hours post infection in a 3.5-hour calcein release assay using an effector∶target ratio of 4. Fibroblasts that were not infected (n.i.) or peptide-loaded (n.i. +peptide) were negative and positive controls, respectively. Data are shown as mean+SD of three to four replicates.

To study the impact of individual HCMV immunovasins on CD8+ T cell killing of infected cells, we employed HCMV strain AD169 and its immunoevasin gene deletion variants CMV-Δall, CMV-US2 and CMV-US11 (expressing only US2 or US11, respectively, but not the other three of the standard set of immunoevasins). Killing of infected cells by CD8+ T cells was subject to the same allotype-specific patterns of immunoevasion as was cytokine secretion (Figure 6), irrespective of pretreatment of the infected cells with IFN-γ. Killing by pp65-specific T cells was not subject to strong immunoevasion, presumably because virion-mediated antigen transfer led to presentation before immunoevasins became active, as described above. HLA-A*0201-restricted killing by IE-1-specific T cells was fully suppressed by the combined HCMV immunoevasins, and largely so by each of the individual proteins US2 or US11, in accordance with the results obtained for cytokine secretion. In contrast, IE-1-specific, HLA-C*0702-restricted killing was less than 50% impaired by the assembled immunoevasins, and not at all by US2 or US11 individually.

### Recognition of C*0702-presented antigen after infection with endotheliotropic HCMV

**Figure 7:**
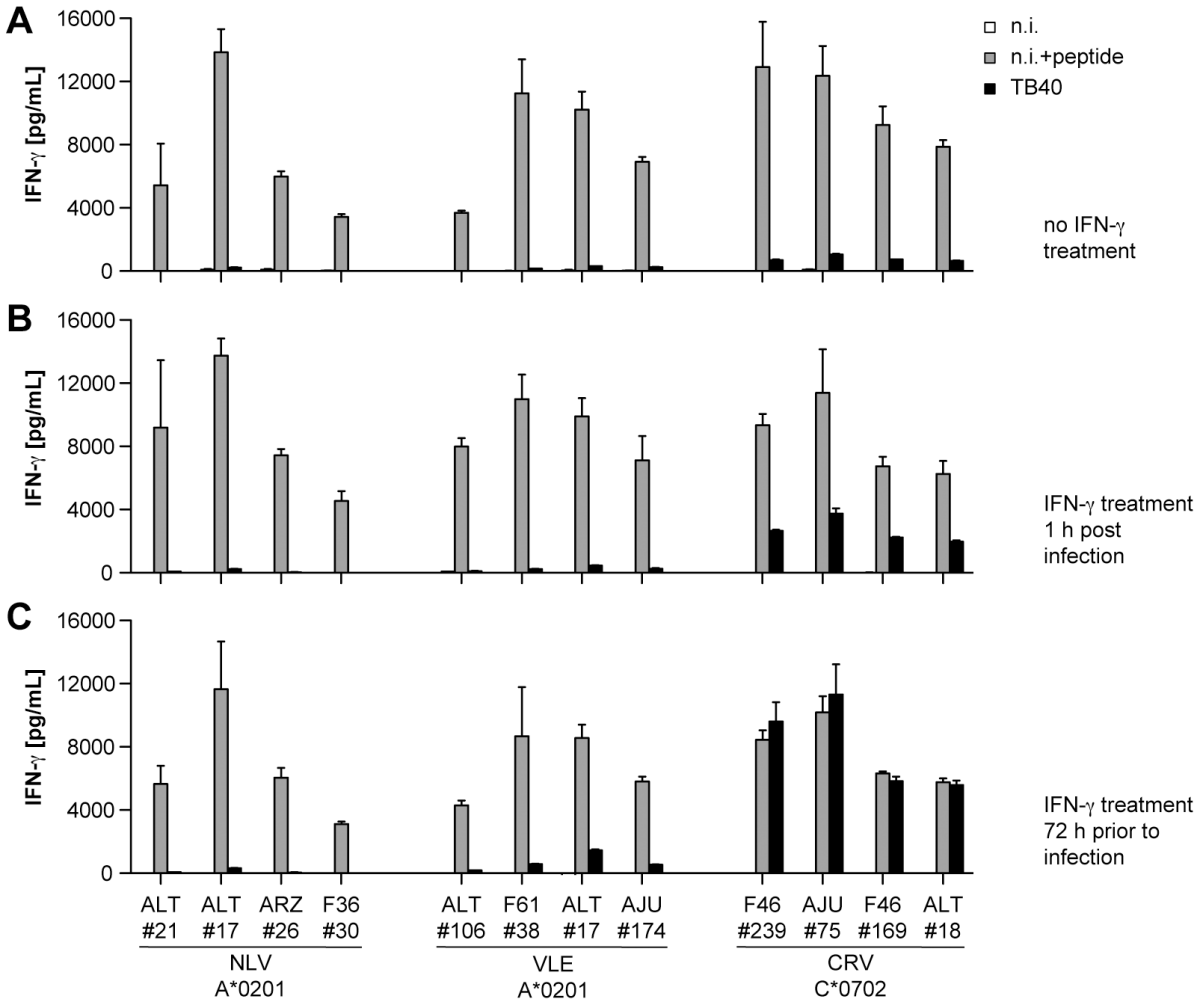
Presentation of IE-1 to CD8+ T cells after infection with endotheliotropic HCMV. MRC-5 fibroblasts were infected with HCMV TB40-BAC4, not infected (n.i.) or peptide-loaded (n.i.+peptide), and their recognition by T cell clones specific for the pp65 epitope NLV/A*0201 and the IE-1 epitopes CRV/C*0702 and VLE/A*0201 was analyzed. Fibroblasts were cultured in standard medium alone (A), with IFN-γ from 1 hour after infection (B), or were pretreated with IFN-γ 72 hours before infection (C). Infection was performed at moi = 5, and effector assays were set up 48 hours post infection (10 000 fibroblasts and 20 000 T cells per well). IFN-γ secretion was measured by ELISA. Data are shown as mean+SD of triplicate samples.

So far, our analyses of antigen presentation by infected cells had employed an established laboratory strain of HCMV, AD169, and its derivatives. However, there are a number of genetic differences between AD169 and low-passage clinical isolates of HCMV [41], and this raised the question whether AD169 was fully representative of wild-type strains in terms of antigen presentation. Of particular relevance, higher expression of the tegument protein pp65 by AD169 than by low-passage isolates [42] could be suspected to affect antigen presentation by competition of pp65 epitopes for certain MHC molecules, or due to other potential immunoevasive functions of pp65 [43], [44]. To address these concerns, we performed T cell recognition experiments with TB40-BAC4, a cloned derivative of endotheliotropic strain TB40E, an HCMV strain that was described to be a valid representative of clinical strains [45]. Of note, TB40-BAC4 lacks the US2-6 region, which was deleted in the course of cloning TB40E as a bacterial artificial chromosome; thus, only the US11 immunoevasin is expressed by this virus. Recognition experiments were performed 2 days after infection with TB40-BAC4 (moi = 5), under three different conditions of pretreatment with IFN-γ, which was added from 72 hours before infection, from 1 hour after infection, or not added at any time (Fig. 7). Strikingly, recognition of the pp65 epitope NLV/A*0201 was fully abolished after infection with TB40-BAC4 under each condition, demonstrating that a single immunoevasin, US11, is sufficient for full repression of pp65 presentation by a recombinant clinical HCMV strain. For the IE-1 epitopes VLE/A*0201 and CRV/C*0702, our earlier experiments with AD169 derivatives were confirmed: presentation of the VLE/A*0201 epitope was strongly suppressed after TB40-BAC4 infection although only US11 was present, whereas CRV/C*0702 presentation was present, and was fully unimpaired in IFN-γ-pretreated cells. Thus, allotype-specific immunoevasion of IE-1 is not limited to a particular HCMV strain, and the higher levels of pp65 in strain AD169 are unlikely to suppress the presentation of IE-1 epitopes, which is in agreement with earlier demonstrations that pp65 does not suppress recognition of IE-1 [21], [24]. Moreover, pp65 is presented by a recombinant HCMV field strain with unexpectedly low efficiency. The experiment also demonstrates that the variant CRVLCCYIL present in some HCMV strains (such as TB40E, Toledo, Davis) is as efficiently presented as the CRVLCCYVL variant present in other strains (such as AD169, Merlin, Towne, strain W), and the same T cell clones recognize both variants. There is little variation in the US11 protein sequence among HCMV strains: only two amino acids are not identical among the four strains Merlin, Toledo, AD169 and TB40E (S42 in AD169, P42 in Merlin, Toledo, and TB40E; I102 in Merlin, V102 in AD169, Merlin and Toledo). Similarly, the US2 sequences of Merlin and Toledo differ from AD169 US2 only in a substitution (E46Q) affecting an amino acid that does not interact with the HLA heavy chain [46]. Therefore, it is likely that the immunoevasins of other HCMV strains have an allotype-specific function that is similar to the viruses AD169 and TB40-BAC4 that were experimentally tested here, and that CRV-specific T cells recognize HCMV IE-1 irrespective of strain variations.

As a further test for a potential interfering role of pp65, we compared the presentation of HCMV epitopes after infection with AD169 wild-type virus and with its pp65-deleted derivative [47]. Deletion of pp65 had no impact on the differential recognition of the IE-1 epitopes VLE/A*0201 and CRV/C*0702 (Figure S1), ruling out a role for pp65 in allotype-specific immunoevasion of IE-1 epitopes in accordance with earlier observations [21], [24].

### Antigen presentation and viral destabilization are mediated by separate domains of HLA class I

**Figure 8:**
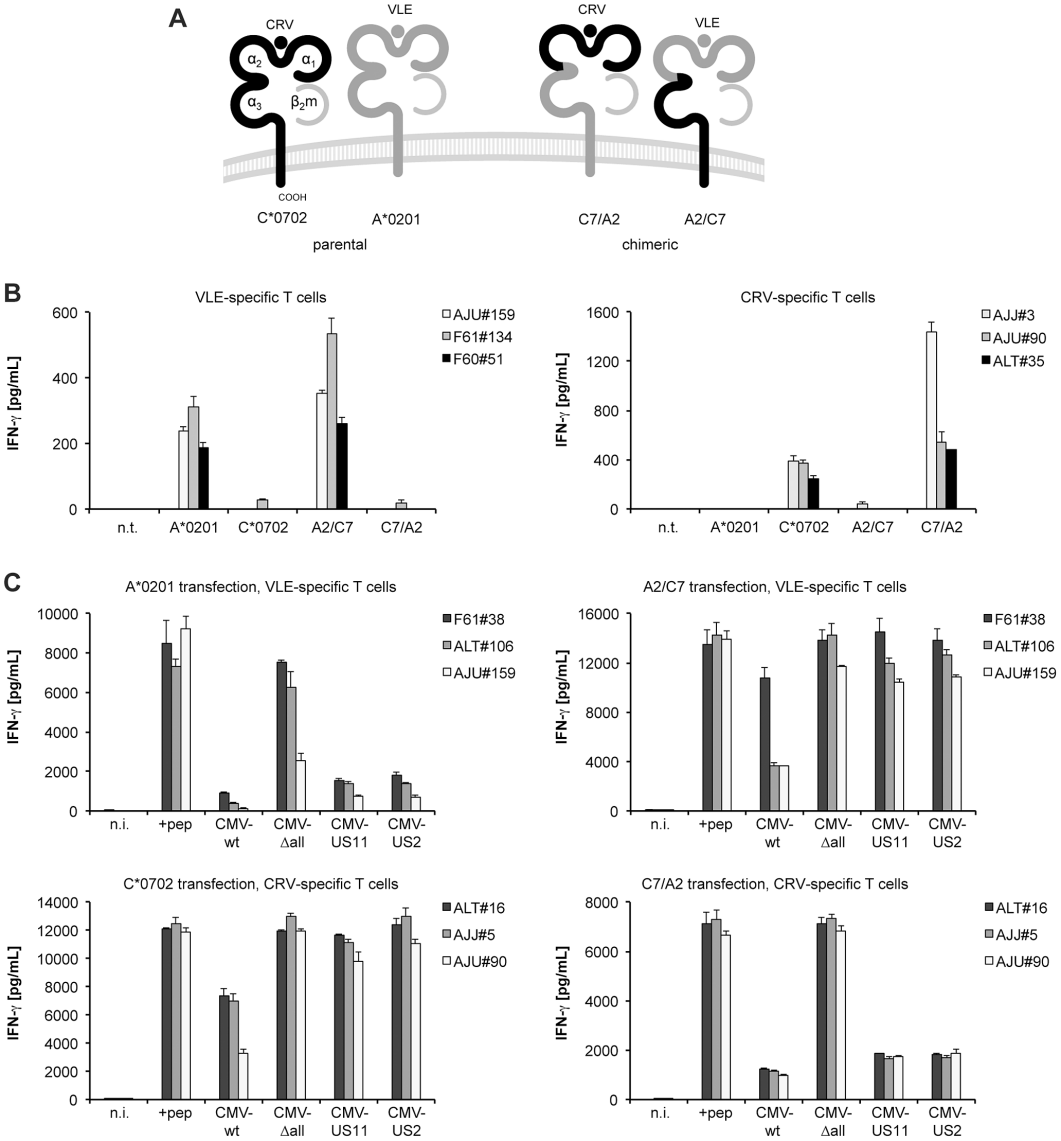
Functional separation of epitope presentation and HCMV immunoevasion. WI-38 fibroblasts (negative for HLA-A*0201 and C*0702) were transfected with plasmids encoding HLA-A*0201, HLA-C*0702, or chimeric HLA class I heavy chains (HLA-A2/C7 or HLA-C7/A2) and subsequently infected with MVA-IE-1 or different HCMV derivatives. IFN-γ secretion was measured in ELISA after overnight incubation of 10 000 clonal T cells with 10 000 target cells. (A) Schematic representation of the native and chimeric HLA class I molecules that were tested. (B) HLA-transfected WI-38 cells were infected with MVA-IE-1, and presentation of IE-1 epitopes was detected by VLE- and CRV-specific T cell clones. Mean and SD of three replicates are shown for 3 T cell clones generated from 3 different donors for each specificity. (C) HLA-transfected WI-38 were infected with CMV-wt, CMV-Δall, CMV-US2 or CMV-US11, not infected (n.i.) or peptide-loaded (+pep). Three T cell clones generated from 3 different donors were used as effectors for each specificity. Data are shown as mean+SD of triplicate samples from one of two independent experiments.

Our T cell experiments showed that HLA-C*0702 and HLA-A*0201 were functionally affected to a very different degree by US2 and US11. These viral glycoproteins interact with different domains of the MHC heavy chains studied so far: US2 with amino acids near the boundary of the α2 and α3 domains, US11 with regions in the α1/α2 domains and, more importantly, in the cytosolic tail of the HLA molecule [48], [49]. We wished to assess whether antigen presentation to CD8+ T cells and destabilizing interactions with HCMV immunoevasins were separable properties of these two extreme instances of HLA class I molecules, HLA-C*0702 and HLA-A*0201, and whether immunoevasion was independent of the presented peptide. To this purpose, we constructed chimeric HLA heavy chains (Figure 8A) that contained the N-terminal part of one of these HLA allomorphs (comprising the α1/α2 domains that bind and present peptide) and the C-terminal part of the other allomorph (comprising the α3 domain, transmembrane region and cytosolic tail). WI-38 fibroblasts, which are negative for HLA-A*0201 and -C*0702, were transfected with constructs encoding parental or chimeric HLA, infected with MVA-IE-1, and tested for recognition by IE-1-specific T cells (Figure 8B). The HLA-A*0201-restricted VLE peptide was only recognized on HLA-A*0201 and chimeric HLA-A2/C7, whereas the C*0702-restricted CRV peptide was only recognized on HLA-C*0702 and chimeric HLA-C7/A2, confirming that the chimeric molecules fully and specifically presented their cognate antigen. We then tested the effects of US2 and US11 on antigen presentation by native and chimeric HLA molecules in the context of HCMV infection (Figure 8C). As seen before, T cell recognition of HLA-A*0201 and HLA-C*0702 epitopes was differentially affected by different HCMV strains in cells transfected with native MHC class I genes: recognition of VLE/A*0201 was strongly reduced by wild-type CMV, CMV-US2, or CMV-US11, whereas recognition of CRV/C*0702 was not impaired by CMV-US2 or CMV-US11 and only partially by wild-type CMV infection. Strikingly, in the chimeric A2/C7 or C7/A2 molecules a complete exchange of the patterns of sensitivity to HCMV immunoevasins was observed. Thus, while the α1/α2 domains present antigen to T cells, the C-terminal part including the α3 domain largely governs the sensitivity of MHC class I to HCMV immunoevasins, and these functions are separable and exchangeable. In addition, this experiment showed that the differences in epitope presentation by HLA-C*0702 and HLA-A*0201 were not caused by different processing efficiencies of the bound peptides, in contrast to previous observations concerning MCMV epitopes [50].

### HCMV-infected fibroblasts inhibit HLA-C*0702-sensitive NK cells

**Figure 9:**
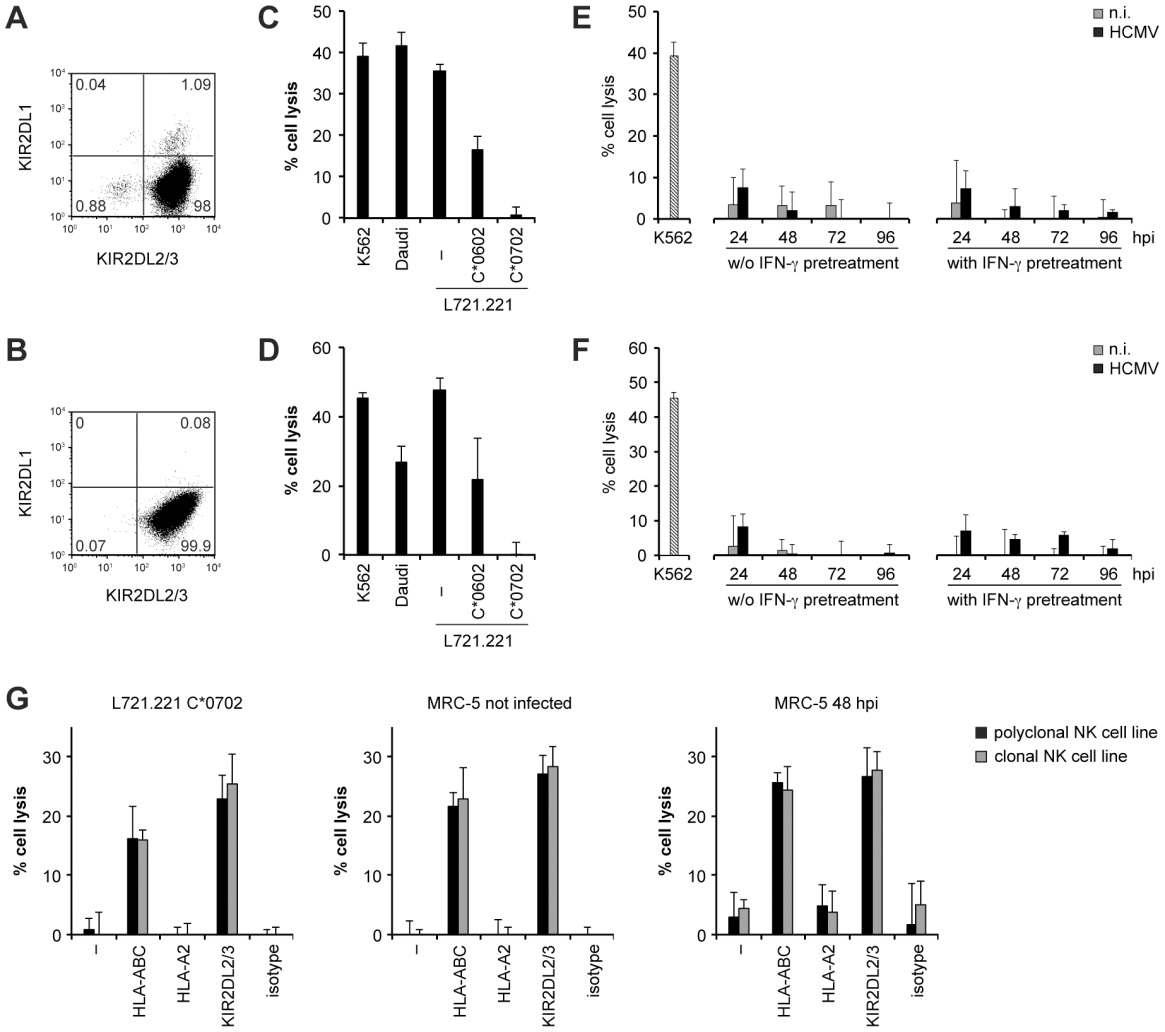
HLA-mediated inhibition of NK cell recognition of HCMV infection. Experiments were performed with polyclonal NK cells (A, C, E, G) or NK cell clone #29 (B, D, F, G) from donor AJU. (A, B) Analysis of KIR expression by flow cytometry. (C, D) Killing by NK cells of the MHC class I-deficient cell lines K562, Daudi and L721.221, HLA-C*0602 or C*0702-transfected L721.221 cells, uninfected MRC-5 fibroblasts, or MRC-5 infected with CMV-wt at moi = 5, at an effector∶target ratio of 2. Data are shown as mean+SD of four replicates from one representative experiment out of four. (E, F) NK cell-mediated killing of uninfected (n.i.) and HCMV-infected fibroblasts over time after infection. Fibroblasts were or were not pretreated with IFN-γ before infection as indicated. (G) Blockade of NK cell mediated-killing by monoclonal antibodies specific for HLA-ABC or KIR2DL2/3 (both IgG2a) or a matched isotype control. Targets were pretreated with IFN-γ. Blockade of the non-KIR ligand HLA-A2 served as additional negative control. The HLA class I type of MRC-5 fibroblasts is HLA-A*0201, A*2902, B*0702, B*4402, C*0501, C*0702. HLA-C*0702 is the only ligand of KIR2DL3 expressed by MRC-5 cells. Killing was assessed at an effector∶target ratio of 2. Data are shown as mean+SD of triplicate samples from one out of two independent experiments.

The selective preservation of antigen presentation by HLA-C*0702 on infected cells was unexpected, because it should facilitate T cell attack *in vivo*. We considered whether the maintenance of HLA-C*0702 conferred an advantage to HCMV. HLA-C allomorphs are ligands for inhibitory killer-cell immunoglobulin-like receptors (KIRs) expressed by NK cells. We tested whether HLA-C*0702 on infected cells prevented their NK cell-mediated killing. We established NK cell lines and clones that uniformly expressed KIR2DL3, a receptor for group 1 HLA-C allotypes including HLA-C*0702. NK cells were obtained from a donor homozygous for group 1 HLA-C alleles to ensure maximal functional competence of KIR2DL3-positive NK cells [51]. Infection experiments were performed in MRC-5 fibroblasts that express HLA-C*0702 as their only KIR2DL3 ligand. Polyclonal NK cells contained 99.1% of KIR2DL3-positive cells; expression of other relevant NK receptors was minor (KIR2DL1+, 1.1%; KIR3DL1+, 3.9%; NKG2A+, 1.7%; Figure 9A). In parallel, NK cell clones were established that were KIR2DL3+ KIR2DL1− KIR3DL1− NKG2A− (Figure 9B). Polyclonal and monoclonal NK cells killed MHC class I-deficient cell lines (K562, Daudi and L721.221) and HLA-C*0602-expressing but not HLA-C*0702-expressing L721.221 cells (Figure 9C, D). Accordingly, blocking of HLA-C*0702 on transfected L721.221 cells with anti-HLA-ABC antibody restored NK cell-mediated lysis (Figure 9G). Killing of HCMV-infected or uninfected MRC-5 fibroblasts was low, irrespective of their IFN-γ pretreatment (Figure 9E, F), suggesting that an inhibitory ligand was present on these target cells. Blocking of HLA-ABC on fibroblasts or of KIR2DL3 on NK cells induced killing of uninfected and HCMV-infected fibroblasts (Figure 9G). These results indicate that HLA-C*0702, the only ligand of KIR2DL3 present in this system, prevented an attack on HCMV-infected fibroblasts by NK cells expressing this inhibitory receptor. Presumably, HCMV preserves HLA-C*0702 on infected cells in order to evade killing by NK cells.

## Discussion

Here we show that MHC class I allotype-specific immunoevasion by HCMV strongly influences CD8+ T cell recognition of infected cells. In particular, IE-1-specific CD8+ T cells that are restricted through a frequent HLA-C allotype, HLA-C*0702, recognize infected cells much more efficiently than HLA-A- and HLA-B-restricted T cells, and presentation of their target epitope resists HCMV immunoevasion in an allotype-specific manner. At the same time, infected cells were resistant to NK cells carrying KIR2DL3, an inhibitory receptor specific for certain HLA-C allotypes including C*0702. These results prompt us to speculate that a requirement to balance first-line defense by NK cells and long-term control by HLA-A- or HLA-B-restricted CD8+ T cells may have influenced the evolution of a complex repertoire of allotype-specific HCMV immunoevasins. As a result, certain HLA-C-restricted CD8+ T cells have particularly efficient antiviral function. Such CD8+ T cells dominated the response to IE-1 in most carriers of this HLA-C allele, suggesting that they may be particularly useful in immunotherapy.

Relationships between immunodominance, epitope processing and epitope presentation in the face of viral immunoevasion have been previously studied in the mouse model. Murine CMV elicits CD8+ T cell responses against two epitopes from the antiapoptotic protein M45, an immunodominant D^b^-restricted epitope and a subdominant D^d^-restricted epitope. While presentation of the dominant epitope is fully abolished in the presence of viral immunoevasins, leading to non-protectivity of the corresponding D^b^-restricted specific T cells in vivo [52], the subdominant epitope from the same antigen is presented more efficiently in the presence of viral immunoevasins and mediates protection in vivo [50]. Analyses of the amounts of intracellularly retained peptides led to the conclusion that the D^d^ peptide was more efficiently processed than the D^b^ peptide, and this difference in processing was responsible for the better presentation of the D^d^ peptide [50]. Although we could not directly assess the processing efficiency of the different IE-1 epitopes in HCMV-infected cells, our domain-swapping experiments show that not the α1/α2 domains or the peptide bound by them, but the C-terminal part of MHC class I molecules guided allotype-specific suppression of antigen presentation by HCMV immunoevasins. Thus, while it is possible that different IE-1 peptides are processed with different efficiencies, such a differential processing was not responsible for differential presentation of the A*0201/C*0702 pair. Moreover, differential availability of other IE-1 epitopes appears unlikely because all tested IE-1 epitopes were presented with approximately equal, near-maximal efficiency in cells that were infected with immunoevasin-deleted HCMV. Because the IE-1 epitope that is most efficiently presented in the presence of immunoevasins is also the most immunodominant one, HLA-C*0702-restricted IE-1-specific T cells will be easily available or inducible in immunotherapeutic settings. Studies of larger panels of epitopes that include other HCMV antigens will show whether other immunodominant epitopes exist whose presentation is similarly efficient. Infection studies in different cell types other than fibroblasts will provide a more comprehensive picture of HCMV immunoevasion, in order to explain the priming and sustenance of T cells specific for epitopes whose presentation is abolished in fibroblasts. Such future analyses may address, for example, whether antigen presentation by directly infected hematopoietic cells [53] is more resistant to HCMV immunoevasins than antigen presentation by fibroblasts, or whether cross-presentation [54] is required for some epitopes to circumvent immunomodulation by HCMV.

A variety of factors may be important in shaping the immunodominance of certain T cells over others. For example, HLA-A*0201-restricted T cell responses to the pp65 antigen from HCMV are numerically smaller in carriers who additionally express HLA-B*0702, an allotype that drives strong pp65-specific T cell responses [55]. This suggests that allotype-specific competition for antigen at the T cell population level influences the composition of T cell repertoires. One antigen of particular interest in this context will be pp65, considering that we unexpectedly found a very low level of presentation of the HLA-A*0201-restricted pp65 epitope NLV by cells infected with the endotheliotropic HCMV derivative TB40-BAC4. The lower levels of pp65 that are expressed by endotheliotropic strains [42], which are considered more representative of HCMV field strains than strain AD169, may render pp65 presentation susceptible to HCMV immunoevasins, potentially resulting in allotype-specific hierarchies of presentation efficiencies that are similar to the one we observed here for IE-1. Conversely, it is possible that cell types other than fibroblasts will present antigens such as pp65 more efficiently in the presence of HCMV immunoevasins.

The importance of HCMV immunoevasins was recently highlighted by the observation that their homologs promote in vivo evasion of CD8+ T cells in a primate model [56]. It seems reasonable to assume that, in humans, CD8+ T cell evasion of HCMV is strongly affected by the diversity of MHC allotypes. However, previous studies on MHC class I allotype-specific effects of HCMV immunoevasins (see below) have been limited to molecular or phenotypic investigations of transfected or transduced cells. Our study provides the first systematic analysis of MHC class I allotype-specific effects of HCMV immunoevasins on CD8+ T cell recognition of infected cells, employing an extended set of CD8+ T cell clones that recognize peptides from the same HCMV antigen, presented by a variety of different human class I allotypes. Although we did not directly demonstrate that the T cell clones studied by us were representative for T cells of the same specificity in vivo, we consider this very likely, because different T cell clones of the same epitope specificity from different donors consistently displayed the same patterns of antigen recognition and its modulation (for example, for the CRV/C*0702 epitope this was true for 21 tested T cell clones from four donors). Our functional data show that allotype-specific HCMV immunoevasion has strong repercussions on T cell recognition of infected cells, and resolve some contradictions between earlier studies. As a case in point, HLA-C expression was moderately reduced when US3 or US6 [57], but not when US2 or US11 [29] were overexpressed in trophoblasts. Our data show that the action of all these viral immunoevasins, when expressed from the viral genome in infected cells, is not sufficient to abolish antigen presentation by HLA-C*0702, strongly supporting the hypothesis that HLA-C/peptide complexes presented by infected placental cells are likely to be a target of cytotoxic T cells [29], and refuting the notion that viral immunoevasion will prevent such a recognition [57]. Molecular elements of HLA molecules that influence their direct or indirect allotype-specific interaction with HCMV immunoevasins have been studied for US2 [49], US3 [58], [59], and US11 [49]. However, such interactions have often appeared to be guided by rather complex rules. This was particularly the case for US11, where variations both in the cytosolic tail and in the α1/α2 domains of HLA molecules were described to influence their allotype-specific downregulation following complex combinatory patterns [60]. None of these studies addressed whether their observed effects had an impact on T cell recognition. Our data show that such complications can be resolved when the biologically relevant read-out, functional T-cell recognition, is directly studied. Our domain-swapping experiments performed with HLA-A*0201 and C*0702 demonstrated that the α1/α2 domains of HLA molecules mediated antigen recognition, whereas the α3 domain and C-terminal region guided allotype-specific immunoevasion, both for US2 and US11 individually and for US2/3/6/11 in aggregate.

NK cell activity is regulated by MHC class I allele-specific signals. The HLA-C/KIR system represents one of the most general mechanisms of human NK cell control: every HLA-C allotype can provide an inhibitory signal through at least one KIR, and every human KIR haplotype encodes KIRs specific for each of the two existing groups of HLA-C [61]. Although NK cell reactivity to HCMV-infected cells is modulated by additional inhibitory and activating NK receptors that are targeted by a multitude of HCMV proteins [62], our experiments indicate that inhibitory signaling through HLA-C and an inhibitory KIR (KIR2DL3) can protect infected fibroblasts from NK cell recognition. Surprisingly, the idea that the downregulation of classical MHC class I molecules shapes NK cell recognition of HCMV-infected cells is controversial, being supported by some [63]–[65] but contested by other studies [66]–[68]. Allotype-specific downregulation of MHC class I can explain such contrasting interpretations. For example, Falk et al. [63] demonstrated that NK lysis was induced after fibroblast infection with wild-type but not US2-11 deleted HCMV. Because the relevant receptor-ligand pair in this study was KIR2DL2 and (likely) HLA-C*0701, this rises the interesting possibility that the two major HLA-C*07 allotypes are differentially regulated by HCMV infection, with different effects on NK cell recognition in carriers of different alleles. Others suggested that KIRs and MHC class I do not play a role in NK recognition of HCMV-infected fibroblasts [68], but did not address the role of individual KIR-HLA pairings in the context of infection. In this context, the enigmatic observation that a majority of NK cell clones from two donors had indifferent reactivity to non-infected and infected autologous fibroblasts [66] can now be explained by the fact that both donors happened to be carriers of HLA-C*0702, leading to HLA-C*0702-mediated inhibition of NK cell reactivity via KIR2DL3 as demonstrated in our study. Taken together, the degree of downregulation of HLA-C allotypes by HCMV may be an important factor in modulating the activity of NK cells in response to infection in a particular carrier.

The present data exemplify the importance of HLA-C in presentation of viral antigens and its important contribution to antiviral T cell repertoires. A special antiviral role of HLA-C due to allotype-specific immunoevasion was previously shown for HIV: the selective downregulation of HLA-A and B, but not C, protects HIV-infected cells against NK cells but sensitizes them to HLA-C-restricted CD8+ T cells [69], [70], and genotypic data showed that certain HLA-C alleles are associated with better control of HIV [71]. Analogously, certain HLA allotypes could potentially predispose to an improved control of HCMV infection. The role of HLA-C-restricted CD8+ T cells in prevention of congenital HCMV infection and pregnancy loss could be of particular importance [29], because placental cells express HLA-C, but not HLA-A or -B [57]. Hypothetically, the existence of an HLA-C*0702-restricted HCMV epitope that mediates superior recognition of infection might be one of the factors that contribute to selection for the high frequency of this allotype in different human populations around the world [29], in whom it represents the most frequent HLA-C allotype with phenotypic frequencies of 30–40% [33], [72]. Further studies may show whether particular MHC class I allotypes or certain HLA/KIR combinations provide a selective advantage due to better defense against the ubiquitous pathogen HCMV, and whether HLA-C-restricted T cells will prove to be particularly efficient effectors in immunotherapy.

## Materials and Methods

### Ethics statement

Mononuclear cells from standard blood donations by anonymous healthy adult donors were obtained from the Institute for Transfusion Medicine, University of Ulm, Germany. The institutional review board (Ethikkommission, Klinikum der Universität München, Grosshadern, Munich, Germany) approved this procedure. All work was conducted according to the principles expressed in the Helsinki Declaration.

### Cells and peptides

HLA typing of blood donations was performed by PCR-based methods (IMGM, Martinsried, Germany). HLA-A, -B and -C alleles and HCMV IgG serostatus (Max von Pettenkofer Institute, Munich, Germany) of the donors are listed in Table S1. Standard cell culture and PBMC preparation was performed as described [73]. Mini-lymphoblastoid cell lines (mLCLs) stably expressing HCMV IE-1, pp65 or no heterologous antigen were generated by infection of PBMCs with B-cell transforming mini-Epstein-Barr virus (mini-EBV) [30]. CD40-stimulated B cell cultures were established and maintained as described [74]. Human fetal lung fibroblast lines MRC-5 and WI-38 were obtained from the European Collection of Animal Cell Cultures (ECACC). BFF2 is a primary human foreskin fibroblast line. Their HLA class I types are as follows: MRC-5, HLA-A*0201, A*2902, B*0702, B*4402, C*0501, C*0702; WI-38, A*0205, A*6801, B*0801, B*5801, C*0701; BFF2, A*0201, A*0301, B*3501, B*4001, C*0304, C*1502. L721.221 cells and their derivatives stably transfected with HLA-C*0602 or -C*0702 [75] were kindly provided by Elfriede Nößner (Munich). K562 and Daudi cells were from the American Type Culture Collection (ATCC).

Peptides were synthesized to >70% purity by JPT (Berlin), resuspended in 100% dimethyl sulfoxide (DMSO) and stored at −20°C. DMSO concentration in all T cell effector assays was kept below 0.1% (vol/vol). The frequency and specificity of IE-1-specific T cells was analyzed by using a library of 120 peptides, each 15 amino acids in length, spanning the entire IE-1 sequence of HCMV strain AD169, with subsequent peptides overlapping in 11 amino acids.

### Recombinant and chimeric MHC class I molecules

HLA-A*0201, -B*0702 and -C*0702 sequences were amplified by PCR from cDNA prepared from HLA-typed LCLs. PCR products were cloned into the vector pCMVcyto (Invitrogen). Plasmids pCMV-HLA-A*0201 and pCMV-HLA-C*0702 were used for the construction of the HLA chimera HLA-A2/C7 (aa −24 to 175 of HLA-A*0201 and 176 to 342 of HLA-C*0702) and its counterpart HLA-C7/A2 (aa −24 to 175 of HLA-C*0702 and 176 to 341 of HLA-A*0201). These switched chimeras were constructed using an Esp3I restriction site in a conserved region near the C-terminal end of the α2-domain.

Fibroblasts were transfected with HLA-encoding plasmids using the Amaxa Cell Line Nucleofector Kit R and Nucleofector I Device (program V-01) (Lonza). Plasmid pEGFP-C1 (BD Biosciences) was used as a transfection control and demonstrated transfection rates of 60–70%. After 24 hours, 200 µg/mL G-418 were added (Invitrogen). Fibroblasts were infected with MVA or HCMV on day 4 and used in T cell effector assays 24 or 48 hours later.

### Viruses

Modified vaccinia virus Ankara (MVA) recombinants expressing either pp65, IE-1 or no HCMV antigen were kindly provided by Naeem Khan (Birmingham, UK) and propagated on baby hamster kidney (BHK-21) cells [12]. For T-cell assays with MHC class I-transfected fibroblasts, cells were infected with MVAs at ten 50% infectious tissue culture dose (TCID_50_) units per cell for 24 hours.

HCMV strain AD169 was kindly provided by Martin Messerle (Hannover, Germany), TB40-BAC4 [45] by Barbara Adler (Munich, Germany). AD169 mutant viruses that lacked the four immunoevasins US2, US3, US6, and US11 (CMV-Δall = RV-KB6) or expressed only one of them, only US11 (CMV-US11 = RV-KB9, deletion of US2, US3 and US6), only US2 (CMV-US2 = RV-KB13, deletion of US3, US6 and US11), only US3 (CMV-US3) or only US6 (CMV-US6), were generated by BAC mutagenesis by successive deletion of individual coding sequences [37], [40]. The AD169 mutant deleted for pp65 (CMV-Δpp65 = RVAd65) was described previously [47]. Stocks of HCMV strains and mutants were prepared by infection of semiconfluent MRC-5 cells at an moi of 0.1. After 14–18 days, supernatants were harvested, cleared from cellular debris by centrifugation, aliquoted and stored at −80°C. Virus titers were determined by infection of MRC-5 cells in flat-bottom 96-well plates at limiting dilution. For T cell effector assays, fibroblasts were precultivated with 300 U/ml recombinant human IFN-γ (PAN Biotech) for 72 hours before infection, cultivated with IFN-γ 1 hour after infection, or not treated with IFN-γ as indicated. Infection with all HCMVs was performed at an moi of 5 for 48 hours, unless indicated otherwise.

### T cells

IE-1- or pp65-specific T cell lines were prepared by restimulation of PBMCs from HCMV-seropositive donors with irradiated (50 Gy) autologous mLCLs expressing the appropriate HCMV antigen [31]. T cell clones were obtained by limiting dilution of these T cell lines or, in some cases, directly from PBMCs after peptide stimulation and IFN-γ secretion assay (Miltenyi Biotec). T cells (0.7 or 2.5 cells/well) were seeded into round-bottom 96-well plates (200 µL/well) in medium supplemented with 1000 U/mL rIL-2, 1×10^5^/mL irradiated (50 Gy) HLA-matched pp65- or IE-1-expressing mLCLs and 1.5×10^6^/mL of a mixture of irradiated (50 Gy) allogeneic PBMCs from at least three different donors. Outgrowing T cell clones were expanded in round-bottom 96-well plates by restimulating every 2 weeks under the same conditions. The T cell clones were specific for the following epitopes: NLVPMVATV (pp65, A*0201) [76], [77], TPRVTGGGAM (pp65, B*0702) [34], [77], ATTFLQTMLR (IE-1, A*6801) [35], KEVNSQLSL (IE-1, B*4001) [35], QIKVRVDMV (IE-1, B*0801) [10], VLEETSVML (IE-1, A*0201) [21], CRVLCCYVL (IE-1, C*0702) and RIKEHMLKK (IE-1, A*0301) (unpublished).

### T-cell effector assays

PBMCs were analyzed for specific IFN-γ secretion in ELISpot, T cell clones in ELISA and cytolysis assays. In any of these assays, antigenic peptides were used whenever indicated at final concentrations of 5 µg/mL per peptide when using single peptides or subpools of up to 12 peptides, and 0.5 µg/mL/peptide when using the complete pool of 129 IE-1 peptides. IFN-γ ELISpot analyses (Mabtech, Nacka, Sweden) were performed in 96-well MultiScreen HTC Filter Plates (Millipore). After antibody coating of the wells, 200 000 PBMCs were distributed to each well, directly loaded with antigenic peptide, and incubated in a total of 200 µL medium per well for 16–18 hours at 37°C and 5% CO_2_. After counterstaining with biotinylated secondary antibody and streptavidin-AP, spots were developed using the AP Conjugate Substrate Kit from Bio-Rad and visually counted after scanning.

To quantify IFN-γ secretion by T cell clones as a measure of antigen recognition [73], effector cells (1–2×10^4^/well, as indicated) were cocultivated for 16–18 h with target cells (1–5×10^4^/well, as indicated) in 200 µL/well of V-bottom 96-well plates at 37°C and 5% CO_2_. Supernatants were harvested and IFN-γ ELISA was performed (Mabtech, Nacka, Sweden).

Lysis of HCMV-infected fibroblasts by CD8+ T cell clones was analyzed by calcein-release assay [73]. Infected or control fibroblasts were washed with PBS and detached by trypsinization. Target cells (1–2×10^6^) were labeled with 5 µg/mL calcein acetoxymethylester (Invitrogen) in 500 µL medium for 30 minutes at 37°C. After washing three times with PBS, 5000 targets/well were co-incubated with 20 000 clonal T cells/well or 40 000 polyclonal T cells/well in V-bottom 96-well plates in a total volume of 200 µL/well. For each type of target, spontaneous release (no T cells added, 0% lysis) and maximal release (0.5% of Triton-X100 added, 100% lysis) was determined. After 3.5 hours of incubation at 37°C and 5% CO_2_, 150 µL/well of supernatant was transferred to a flat-bottom 96-well plate, and fluorescence intensity at 485/535 nm (excitation/emission) was measured in a Wallac Victor counter (Perkin-Elmer).

### Flow cytometry

CD8+ T cells specific for the HLA-B*0702-restricted TPR epitope or the HLA-C*0702-restricted CRV epitope were quantified with MHC class I multimer reagents. CRV-specific CD8+ T cells were stained using MHC OneSTrEPtag-*Strep*-Tactin multimers [78]. CRV/HLA-C*0702 OneSTrEPtag monomers were prepared as described [79]. For multimerization, monomers were incubated with PE-labeled *Strep*-Tactin (IBA, Göttingen) for 45 minutes at a 1∶1 molar ratio. Then, 5×10^5^ PBMCs were incubated with an aliquot of the assembled streptamer reagent containing 2.5 µg HLA-C*0702 monomer in a total volume of 30 µl PBS+2% FCS for 30 minutes on ice. Cells were counterstained by adding CD8-APC (BioLegend) to the streptamer staining mixture and incubating for 20 minutes on ice. For quantification of TPR-specific T cells, 5×10^5^ PBMCs were incubated with 1 µL HLA-B*0702/TPR pentamer (Proimmune, Oxford, UK) for 10 minutes at room temperature. After washing with PBS+2% FCS, T cells were counterstained with pentamer-binding Pro5 Fluorotag R-PE (Proimmune) and CD8-APC (BioLegend) for 20 minutes on ice.

To characterize NK cells, the following antibodies were used: CD56-PE-Cy5 (clone HCD56, BioLegend), KIR2DL1-FITC (clone HP-3E4, BD Pharmingen), KIR2DL2/3-PE (clone CH-L, BD Pharmingen), KIR3DL1-FITC (clone DX9, BioLegend), NKG2A-APC (clone 131411, Beckman Coulter).

After staining, cells were washed with PBS+2% FCS and fixed with 1% formaldehyde (Carl Roth). Cells were analyzed on a BD Biosciences FACSCalibur flow cytometer. Data analysis was performed using FlowJo 9.4.11 software (Tree Star).

### NK cells

Donor AJU was positive for KIRs 2DL1, 2DL3, 2DL4, 2DS4, 3DL1, 3DL2, 3DL3, and negative for KIRs 2DL2, 2DL5, 2DS1, 2DS2, 2DS3, 2DS5, 3DS1 (IMGM, Martinsried, Germany). KIR2DL3-expressing NK cells from PBMCs of donor AJU were enriched by immunomagnetic depletion of CD3+ cells with CD3 MicroBeads (Miltenyi Biotec) followed by positive isolation using PE-labeled anti-KIR2DL2/3 antibody (clone CH-L, BD Pharmingen) and anti-PE MicroBeads (Miltenyi). KIR2DL3-enriched NK cells were expanded in bulk and, in parallel, under conditions of limiting dilution. In both cases, NK cells were cultivated in round-bottom 96-well plates (200 µL/well) in standard cell culture medium supplemented with 500 U/mL rIL-2 and restimulated every two weeks with a feeder mixture consisting of 2×10^5^/mL irradiated (50 Gy) HLA-C1 group-positive allogeneic mLCLs and 1×10^6^/mL irradiated (50 Gy) allogeneic PBMCs from at least three different donors.

Killing by NK cells was analyzed by calcein-release assay [73] at an effector/target ratio of 2/1. To mask MHC class I molecules on target cells, purified HLA-ABC- (W6/32, IgG2a) or HLA-A2- (BB7.2, IgG2b) specific antibodies (BioLegend) were added at 60 µg/mL to the target cells for 1 hour at 37°C prior to addition of effector cells. To inhibit KIR2DL2/3 interaction with its MHC class I ligands, NK cells were preincubated with an antibody blocking KIR2DL2/3 (clone DX27, IgG2a, BioLegend) at 60 µg/mL for 1 hour at 37°C. Purified mouse IgG2a and IgG2b (BioLegend) were used as isotype controls.

## Supporting Information

Table S1 **HLA types and HCMV carrier states of donors.**
(PDF)

Figure S1 **Impact of pp65 on the presentation of IE-1 epitopes by infected cells.** Fibroblasts were pretreated with IFN-γ for three days and infected with CMV strain AD169 (CMV-wt) or an AD169 mutant deleted for the pp65 gene. One day after infection, recognition of infected cells by clonal CD8+ T cells with the indicated specificities was tested in an IFN-γ ELISA at an effector target ratio of 1∶1 (A) and a cytotoxicity assay at an effector-target ratio of 4∶1 (B). Data are shown as mean+SD of triplicate samples.
(TIF)
