# Supplementary material for: Presentation of an Immunodominant Immediate-Early CD8+ T Cell Epitope Resists Human Cytomegalovirus Immunoevasion
Source: PLoS Pathog. 2013 May 23;9(5):e1003383. doi: 10.1371/journal.ppat.1003383 (PMC3662661; doi:10.1371/journal.ppat.1003383)
Supplement: Table S1 — HLA types and HCMV carrier states of donors. (PDF) [file ppat.1003383.s001.pdf]

Table S1. HLA types and HCMV carrier state of donors.

Donor	HLA-A	HLA-B	HLA-C	HCMV serostatus
AAJ	*0201, *0301	*3801, *4403	*0401, *1203	Neg
AES	*2602, --	*3501, *4801	*0401, *0803	Pos
AJJ	*0201, --	*0702, *3503	*0401, *0702	Pos
AJG	*0201, --	*3503, *5701	*04, *06	Pos
AJU	*0201, *2402	*0702, *4001	*0304, *0702	Pos
ALT	*0201, --	*0702, *4002	*0202, *0702	Pos
AMD	*01, *11	*08, *1501	*0303, *0701	Pos
ARZ	*0201, *2902	*4402, *4501	*0602, --	Pos
ASG	*0101, *2902	*0801, *4403	*0701, *1601	Pos
ASI	*0201, --	*44, *51	*02, *05	Neg
ASM	*03, *33	*07, *14	*0702, *0802	Neg
F43	*01, *24	*07, *57	*0602, *0702	Pos
F46	*03, *11	*07, *3501	*0401, *0702	Pos
F59	*02, *6801	*1801, *4001	*0304, *0701	Pos
F60	*02, *26	*07, *38	*0702, *1203	Pos
F61	*02, *34	*14, *44	*0401, *0802	Pos
F63	*03, *24	*18, *4001	*0304, *0701	Pos
F64	*0201, *2902	*1501, *4403	*0303, *1601	Pos
F65	*24, *68	*35, *44	*0401, *1504	Pos
LM02	*03, *24	*07, *18	*0701, *0702	Pos
LM16	*0301, *3101	*0702, *0801	*0701, *0702	Pos
LM20	*0201, *2402	*0702, *3901	*0702, *1203	Pos
LT12	*11, *11	*07, *55	*0102, *0702	Pos
SA01	*0301, *6801	*0702, *5101	*0702, *1502	Pos
SA03	*0101, *0301	*0702, *5701	*0602, *0702	Pos
SA04	*0102, *0201	*0702, *4901	*0701, *0702	Pos
SA12	*01, *03	*07, *35	*0401, *0702	Pos
SA13	*03, --	*07, *52	*0702, *1202	Pos
