# Supplementary figures and images for: Presentation of an Immunodominant Immediate-Early CD8+ T Cell Epitope Resists Human Cytomegalovirus Immunoevasion
Source: PLoS Pathog. 2013 May 23;9(5):e1003383. doi: 10.1371/journal.ppat.1003383 (PMC3662661; doi:10.1371/journal.ppat.1003383)

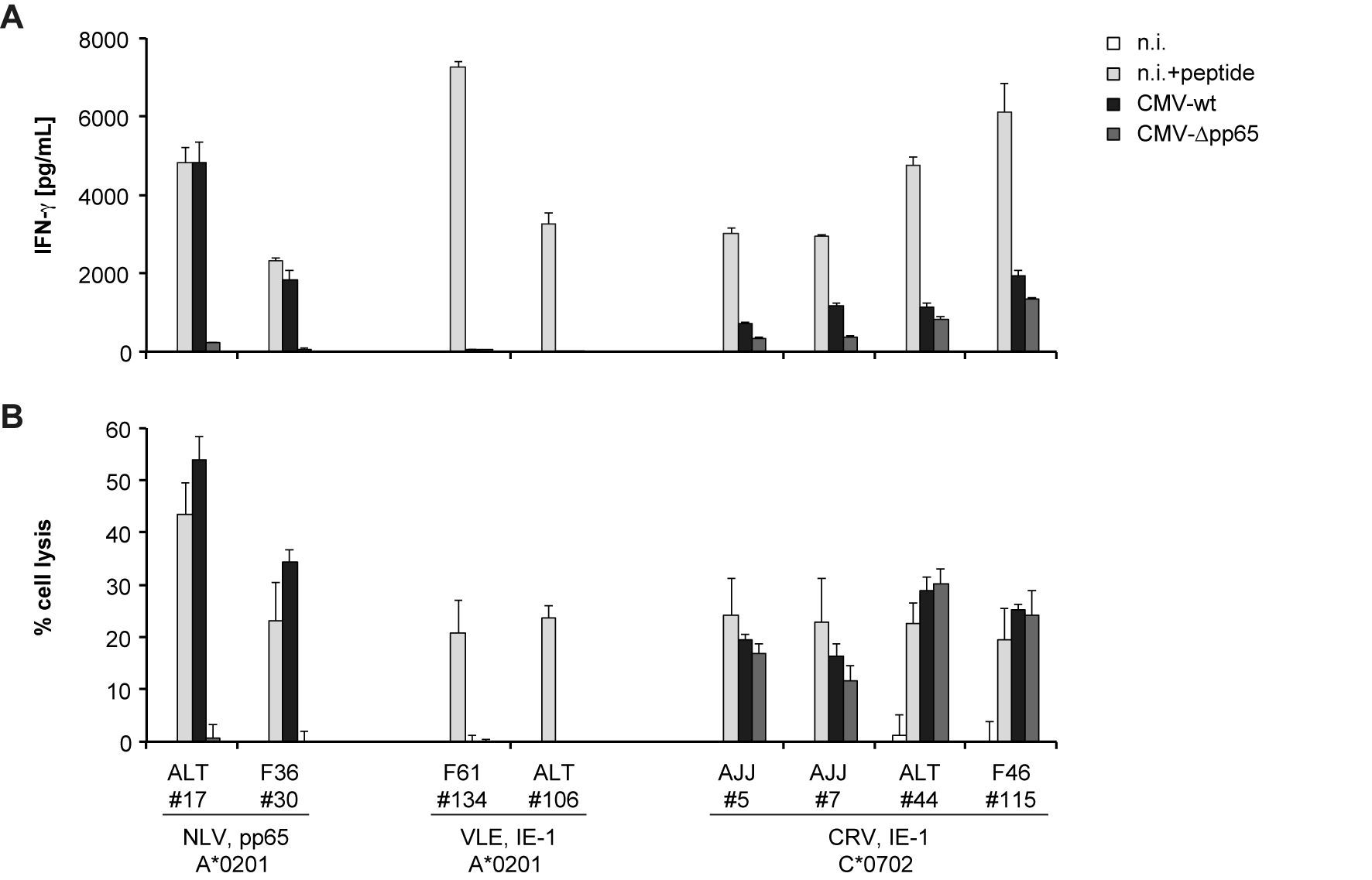

Supplement: Figure S1 — Impact of pp65 on the presentation of IE-1 epitopes by infected cells. Fibroblasts were pretreated with IFN-γ for three days and infected with CMV strain AD169 (CMV-wt) or an AD169 mutant deleted for the pp65 gene. One day after infection, recognition of infected cells by clonal CD8+ T cells with the indicated specificities was tested in an IFN-γ ELISA at an effector target ratio of 1∶1 (A) and a cytotoxicity assay at an effector-target ratio of 4∶1 (B). Data are shown as mean+SD of triplicate samples. (TIF) [file ppat.1003383.s002.tif]
